# Endoluminal Reconstruction of Complex Cervical Internal Carotid Artery Dissections Using the Surpass Elite Flow Diverter: A Technical Report

**DOI:** 10.7759/cureus.117324

**Published:** 2026-10-01

**Authors:** Helio García, Fernando Ahumada, Orlando Diaz

**Affiliations:** 1 Interventional Neuroradiology, Houston Methodist, Houston, USA; 2 Interventional Neuroradiology, Houston Methodist Neurological Institute, Houston, USA

**Keywords:** blunt cerebrovascular injury, carotid artery dissection, digital subtraction angiography, endovascular reconstruction, flow diverter, pseudoaneurysm, surpass elite

## Abstract

Most cervical internal carotid artery (ICA) dissections heal with antithrombotic therapy. Complex lesions with flow-limiting stenosis, multisegment involvement, or persistent wide-necked pseudoaneurysms may fail medical management and require endovascular reconstruction, the indications for which remain individualized. Conventional carotid stents conform poorly to acute curves. Flow diverters reconstruct the parent lumen and accommodate angulation, but cervical use is off-label and experience with current-generation devices is limited. To our knowledge, we report the first use of the Surpass Elite flow diverter (Stryker Neurovascular, Fremont, CA) for cervical ICA dissection, in two patients, as the primary scaffold within hybrid constructs supported by coil embolization, angioplasty, and carotid stenting. An 18-year-old female with a Biffl grade III blunt injury had a right cervical dissection with critical narrowing of the true lumen and two large wide-necked pseudoaneurysms persisting despite antiplatelet therapy; coil embolization of both sacs, a single flow diverter, angioplasty, and telescoped carotid stents restored luminal caliber, with six-month angiography showing complete resolution without coil compaction. A 72-year-old male with a prior left middle cerebral artery infarct had a 6-cm chronic dissection of the cervical left ICA with dense distal calcification, aneurysmal dilatation, 70% stenosis, and angiographic hypoperfusion; two telescoped flow diverters and a carotid stent reconstructed the lumen without residual stenosis. Both patients received dual antiplatelet therapy starting seven days preoperatively, with P2Y12 reaction units verified within the target range, and neither had periprocedural complications. Reconstruction with the Surpass Elite within a hybrid construct was technically feasible in these two contrasting morphologies; two cases cannot establish efficacy, and the independent contribution of the device cannot be determined.

## Introduction

Cervical artery dissection accounts for approximately 2% of all ischemic strokes but for 10-25% of ischemic strokes in adults under 45 years of age [1], and internal carotid artery (ICA) dissection carries a high rate of ischemic events within the first 30 days [2]. Antithrombotic therapy is first-line management, and low-grade intimal injury heals reliably with medical therapy [3,4]. Most dissections therefore never require endovascular intervention.

A subset does not heal. The natural history of dissecting pseudoaneurysms is variable; most remain stable or heal on antithrombotic therapy, whereas a minority persist or enlarge [3,5-7]. Although uncommon, extracranial carotid pseudoaneurysms may rupture with potentially life-threatening hemorrhage [8]. Lesions producing flow-limiting stenosis, extending over several centimeters, or involving more than one segment carry persistent risk of thromboembolism and hemodynamic compromise [3,5]. Indications for reconstruction in these complex extradural ICA dissections remain individualized. Primary coiling fills the sac without treating the flap; stent-assisted coiling can treat flap and sac in a single session when an appropriately sized stent covers the dissected segment; covered stents exclude the lesion but are stiff, require large delivery systems, and occlude covered branches [2,5]. Conventional carotid stents are comparatively rigid and conform poorly through curves, so when a dissection is accompanied by marked tortuosity, the procedure becomes technically demanding.

Flow diverters behave differently. Their low porosity redirects flow along the reconstructed parent lumen, promotes intra-aneurysmal thrombosis, and scaffolds neointimal overgrowth across both neck and flap, while the compliance of a braided construct permits apposition in tortuous anatomy [2,5,9]. The strategy itself is established: reports of flow diversion for iatrogenic and traumatic ICA injury describe high technical success, although the published experience rests on earlier-generation devices [2,5,10,11], and stand-alone flow diversion of cervical dissections has been described with these devices [2,10]. The Surpass Elite (Stryker Neurovascular, Fremont, CA), commercially released in 2025 [12], differs from earlier Surpass generations in braid composition and braid angle [13,14]. Its clinical characterization is currently confined to intracranial cohorts [13], and its use in the cervical carotid has not been reported. Use in this territory is off-label in two respects, as flow diverters are approved for intracranial aneurysms whereas these lesions are extracranial dissections.

The Surpass Elite uses a 64-wire braid (52 cobalt-chromium and 12 platinum-tungsten wires) with full-length radiopacity and a braid angle above 150° [13]. In principle, a higher braid angle helps preserve mesh density at larger diameters and reduces the tendency of the device ends to lift in curved segments; these properties have not been demonstrated in the cervical carotid. Devices of 50 mm length are available up to 5.25 mm in diameter, allowing long segments to be covered with fewer overlapping implants [13,15,16].

We describe two patients in whom this device formed the basis of a reconstruction, chosen because tortuosity made a conformable braided implant preferable to a conventional carotid stent. A search of PubMed, Embase, and Google Scholar on August 10, 2026, using "Surpass Elite" AND (dissection OR cervical OR carotid OR extracranial), with no language, date, or publication-type restrictions, identified no previous report of this device for cervical ICA dissection. To our knowledge, this is the first reported use of the Surpass Elite in this indication in the indexed literature.

## Technical report

Endovascular reconstruction was considered when follow-up imaging showed either a pseudoaneurysm persisting with critical narrowing of the true lumen despite antiplatelet therapy (case 1), or angiographic progression of a flow-limiting stenosis with hypoperfusion in a patient with a prior ipsilateral infarct (case 2).

Case 1: Acute post-traumatic dissection with large wide-necked pseudoaneurysms

An 18-year-old female with no relevant medical history was injured in a motor vehicle collision, sustaining polytrauma, including a displaced femoral shaft fracture, facial fractures requiring plate fixation, sternal contusion, and a shoulder injury (modified Rankin Scale (mRS) score 0). Computed tomography angiography (CTA) demonstrated a right ICA dissection extending to the skull base with high-grade stenosis. Clopidogrel 75 mg orally daily was started and continued as monotherapy until the intervention, approximately 11 weeks after diagnosis. Repeat CTA approximately 10 weeks after diagnosis showed persistent dissection of the right mid-cervical ICA with two large wide-necked pseudoaneurysms and critical narrowing of the true lumen (Biffl grade III) [17]. No ischemic events occurred during this interval, and head CT showed no interval change. Diagnostic angiography confirmed the lesion and showed collateral filling of the right ICA territory, predominantly through a hypertrophic right posterior communicating artery, with minor cross-filling through the anterior communicating artery despite a hypoplastic right A1 segment (Figure 1).

**Figure 1:**
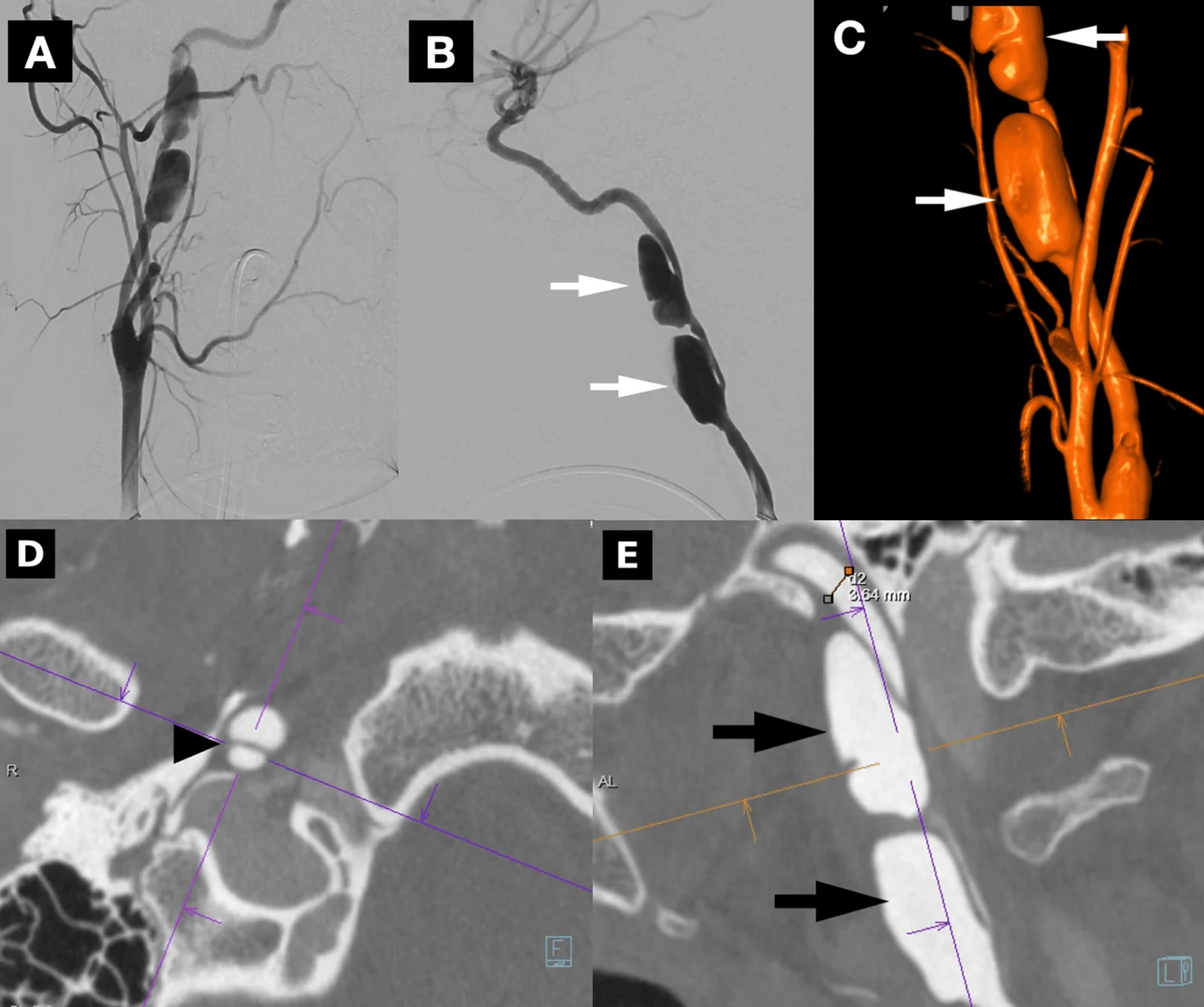
Pre-treatment angiography of the right cervical internal carotid artery dissection. (A) Frontal and (B) lateral projections of a right carotid artery injection showing a complex dissection of the cervical right internal carotid artery with two large pseudoaneurysms (arrows). (C) Three-dimensional rotational reconstruction. (D, E) Cone-beam CT reconstructions showing the double lumen (arrowhead) and both pseudoaneurysms (arrows).

Access and Navigation

Under general anesthesia via a right common femoral approach, a 6-French system guided a Headway 17 microcatheter (MicroVention, Aliso Viejo, CA) into the distal and proximal pseudoaneurysm sacs. True-lumen position was confirmed during crossing by the configuration of a J-shaped wire tip and, after crossing, by cone-beam CT with diluted contrast injection.

Sac Coiling

As flow diverter-induced thrombosis of the sac may take more than three months, platinum coils (Balt, Montmorency, France) were deployed to accelerate sac thrombosis and healing and to reduce the risk of hemorrhage during that interval.

Flow Diverter Deployment

A 5 mm × 50 mm Surpass Elite was deployed from the petrous to the proximal cervical ICA as the primary reconstructive scaffold. Post-dilation angioplasty was performed with a 6 mm × 15 mm Eclipse balloon (Balt, Montmorency, France).

Hybrid Scaffolding

To secure the construct across the mobile-to-fixed transition of the carotid bulb, 5 mm × 40 mm and 7 mm × 40 mm Precise carotid stents (Cordis, Miami Lakes, FL) were telescoped proximally (the "proximal anchoring technique"), with 12 mm of overlap between the Surpass Elite and the Precise construct (Figure 2).

**Figure 2:**
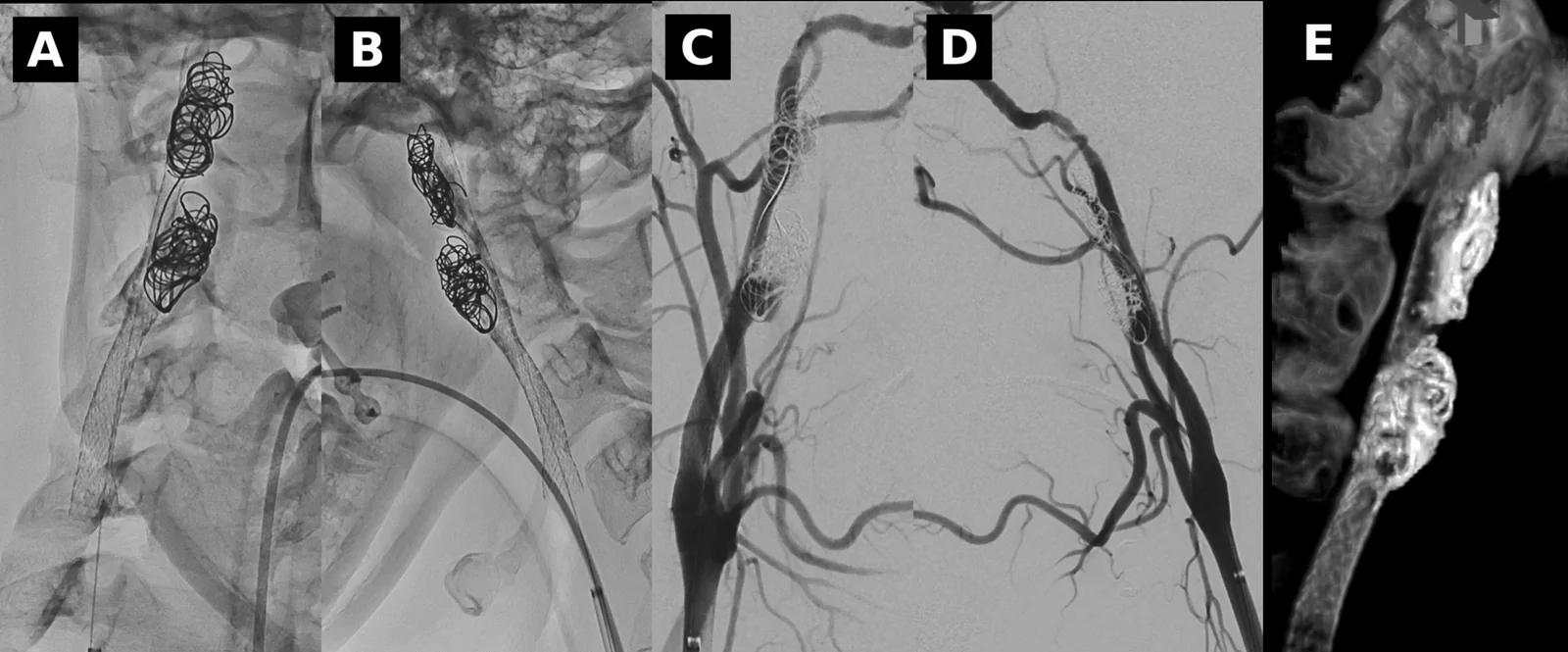
Immediate post-reconstruction angiography (case 1). (A, B) Unsubtracted and (C, D) subtracted right common carotid artery injections showing the Surpass Elite flow diverter and the two telescoped Precise carotid stents reconstructing the lumen of the right internal carotid artery. (E) Cone-beam CT reconstruction showing the reconstructed right internal carotid artery with coils within both pseudoaneurysms.

The patient followed a strict dual antiplatelet therapy (DAPT) protocol: aspirin 81 mg daily was added to clopidogrel 75 mg daily seven days preoperatively, with P2Y12 reaction units (PRU) measured three days before and on the day of the procedure, held between 60 and 180 PRU (105 PRU on procedure day), a target range intended to reduce thromboembolic and hemorrhagic complications [18]. Fluoroscopy time was 44.4 minutes, with a cumulative reference air kerma of 2769 mGy. She awoke without deficit and was discharged on postoperative day 2. Six-month cerebral angiography demonstrated complete restoration of luminal caliber and complete resolution of both pseudoaneurysms without coil compaction or in-stent stenosis (Figure 3).

**Figure 3:**
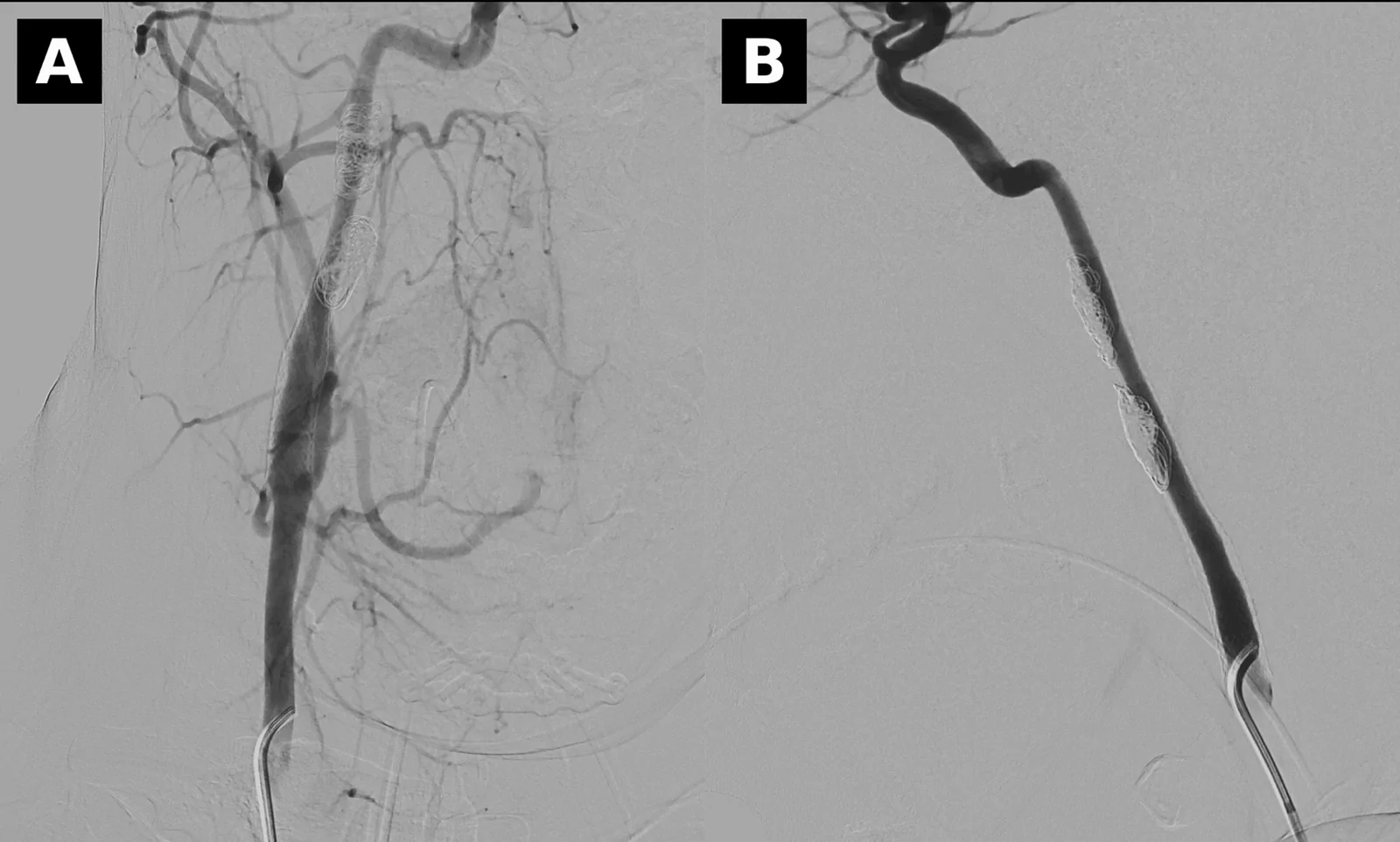
Six-month follow-up angiography (case 1). (A) Frontal projection of a right common carotid artery injection and (B) lateral projection of a right internal carotid artery injection showing complete restoration of the lumen of the right internal carotid artery, with good apposition of the carotid stents and the Surpass Elite flow diverter and no in-stent stenosis.

Case 2: Chronic calcified dissection with flow-limiting stenosis

A 72-year-old male with a remote left middle cerebral artery (MCA) territory infarct, with residual right hemiparesis, aphasia, and mild confusion (mRS 4), and a known chronic left cervical ICA dissection sustained a fall in March 2026, plausibly related to these deficits. CTA obtained after the fall showed irregularity of the left cervical ICA with a focal dissection flap, aneurysmal dilatation, and dense distal calcification. Diagnostic angiography in July 2026 demonstrated interval progression: a 6-cm dissection with 70% stenosis, measured by the North American Symptomatic Carotid Endarterectomy Trial (NASCET) method [19] against the normal distal straight segment of the cervical ICA, flow limitation, and relative hypoperfusion of the left MCA territory, defined angiographically as delayed antegrade opacification of the intracranial circulation without intracranial occlusion or critical stenosis (Figure 4). Reconstruction was performed four weeks later, in August 2026.

**Figure 4:**
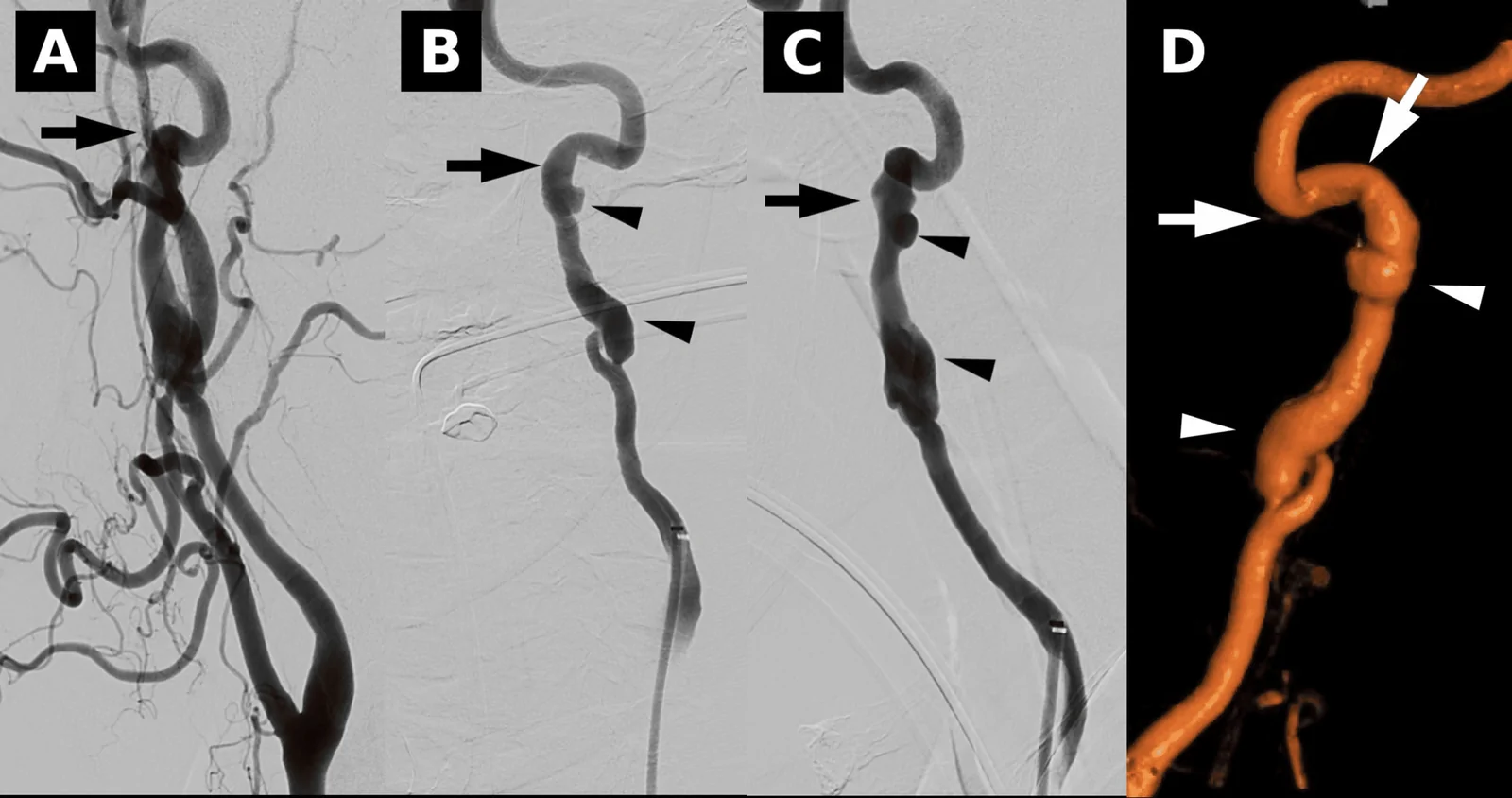
Pre-treatment angiography of the left cervical internal carotid artery dissection (case 2). (A) Lateral projection of a left common carotid artery injection and (B, C) selective left internal carotid artery injections in two projections, with (D) a three-dimensional rotational reconstruction, showing a complex dissection of the cervical left internal carotid artery associated with two small pseudoaneurysms (arrowheads). Note the tortuosity of the distal cervical internal carotid artery, which made conventional carotid stenting unfavorable.

Access and Navigation

A 7-French guiding catheter and a Vecta 46 intermediate catheter (Stryker Neurovascular, Fremont, CA) crossed the 6-cm calcified lesion. An Excelsior XT-27 microcatheter (Stryker Neurovascular, Fremont, CA) reached the cavernous segment.

Flow Diverter Deployment

Two 5.25 mm × 50 mm Surpass Elite devices were telescoped from the petrous segment to the bulbar segment, overlapping by 20 mm. No kinking or wall-gapping was observed at the 90° turn at the petrous transition, as assessed on unsubtracted and subtracted two-dimensional projections and on cone-beam CT reconstructions.

Hybrid Scaffolding and Angioplasty

An 8 mm × 40 mm Precise carotid stent (Cordis, Miami Lakes, FL) was placed proximally across the stenosis to provide radial support. Angioplasty was performed with a 4 mm × 10 mm Scepter compliant balloon (MicroVention, Aliso Viejo, CA) within the Surpass Elite segment and a 4.5 mm × 20 mm Gateway balloon (Stryker Neurovascular, Fremont, CA) within the Precise stent (Figure 5).

**Figure 5:**
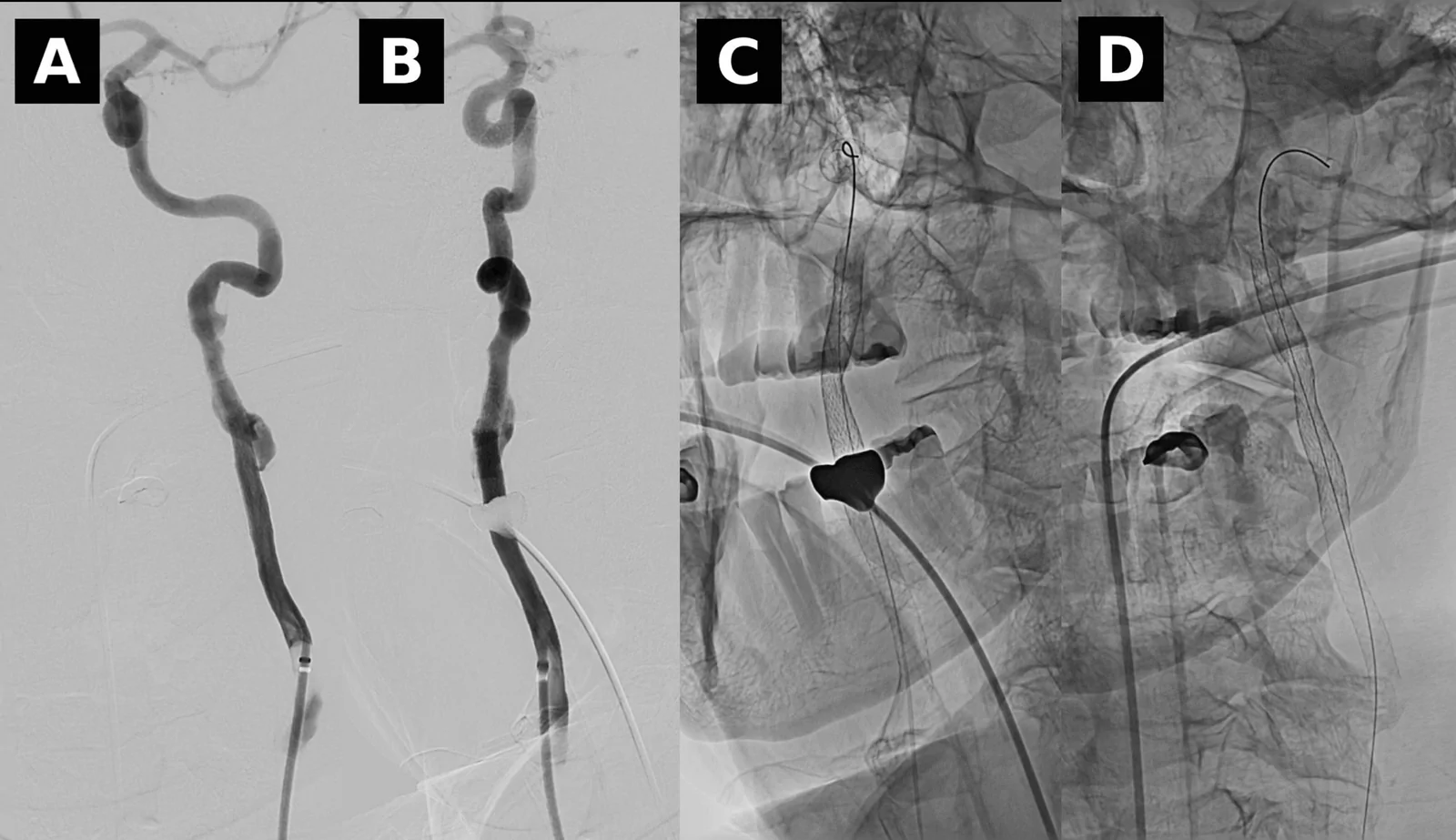
Immediate post-reconstruction imaging (case 2). (A, B) Angiographic images showing the two telescoped Surpass Elite flow diverters and the Precise carotid stent reconstructing the cervical left internal carotid artery. (C, D) Cone-beam CT reconstructions demonstrating the final construct.

DAPT followed the same protocol (99 PRU on procedure day). Fluoroscopy time was 42.2 minutes, with a cumulative reference air kerma of 1114.5 mGy. Immediate angiography confirmed restoration of luminal caliber with appropriate antegrade flow, good construct apposition, and contrast stagnation within the aneurysmal component (Figure 5). He was discharged on postoperative day 2 at his neurological baseline (mRS 4). Six-month angiographic follow-up is scheduled.

## Discussion

Flow diversion has been reported as a reconstructive option for cervical ICA dissections with pseudoaneurysm or flow-limiting stenosis that fail medical therapy, with high technical success using earlier-generation devices [2,5,9-11]. The aim of this report is to describe the technical feasibility of the Surpass Elite, within hybrid constructs, in two anatomically different complex cervical ICA dissections.

These two patients presented opposite morphologies of the same disease, and in each the construct was assembled to answer the specific anatomical problem. When evaluating endovascular options for complex extradural ICA dissections, each conventional modality presents specific trade-offs regarding luminal scaffolding, branch preservation, and conformability (Table 1).

**Table 1:** Endovascular options for complex cervical internal carotid artery dissection.

Option	Mechanism	Principal consideration in complex cervical dissection
Antithrombotic therapy alone	Prevents thromboembolism while the intimal injury heals	Effective for low-grade injury; most pseudoaneurysms remain stable or heal, and flow-limiting stenosis is not addressed [3,5].
Primary coil embolization	Fills the aneurysmal sac	Treats the sac but not the dissection flap; protrusion and migration are concerns in a wide-necked sac within a mobile artery [2,5].
Stent-assisted coiling	Scaffolds the neck during coiling	Can treat flap and sac in a single session when an appropriately sized stent covers the dissected segment [2,5].
Covered stent graft	Excludes the lesion from the circulation	Effective exclusion where apposition can be achieved; stiff, large delivery system, occludes covered branches, and apposition is less predictable in a calcified or angulated segment [2,5].
Flow diversion	Redirects flow along the reconstructed parent lumen; scaffold for neointimal overgrowth across flap and neck	Conforms to tortuous anatomy and treats flap and sac together; approved for intracranial aneurysms, so cervical use for dissection is off-label in both anatomy and indication, and dual antiplatelet therapy is required [2,5,9].

In case 1, the difficulty was the pseudoaneurysm mass and neck geometry across two segments. Coils reduced inflow into both sacs, while the Surpass Elite provided the reconstructive scaffold. The telescoped carotid stents extended scaffolding proximally across the carotid bulb, where a braided device sized for the intracranial circulation is comparatively undersized and susceptible to migration or proximal neointimal reaction driven by neck movement. This layered arrangement corresponds to the proximal anchoring technique described by Campos et al. in 31 petrocervical dissections [5].

Case 2 posed a different problem: lesion length and calcification. A 6-cm lesion with distal calcification made covered stent reconstruction unfavorable due to poor compliance. Two 50 mm devices were telescoped with 20 mm of overlap to cover the length, a configuration permitted by the size matrix of this platform, with a single carotid stent for proximal radial support across the mobile-to-fixed transition. Fluoroscopy time and cumulative reference air kerma are reported for each case; because the procedures differed in content, the values are not directly comparable. Both were below the 5,000 mGy reference point air kerma defined as a significant radiation dose requiring patient follow-up [20].

Device selection was guided by the need for conformability in tortuous anatomy and preserved mesh density at wide calibers. The high braid angle and 64-wire construction [13] were the theoretical rationale for this choice; they are not properties demonstrated by this series. Carotid stents were added proximally because the largest Surpass Elite diameter (5.25 mm) is undersized for the proximal cervical ICA and bulb, and because the bulb is the mobile-to-fixed transition where proximal anchoring has been described [5]. The technical results are congruent with the published experience with earlier-generation devices in comparable anatomy [2,5,9,10].

The antithrombotic requirement is the principal constraint of this approach. Flow-diverter constructs require DAPT to reduce thrombotic complications of the implanted devices, a consideration of particular weight in case 2, in which an elderly patient with a prior stroke was committed to six months of dual therapy. Limitations include the small cohort, the absence of a comparison group, the lack of follow-up imaging in case 2, and the off-label use of extracranial flow diversion. Because both constructs were hybrid, with additional carotid stents, angioplasty, and, in case 1, coil embolization, the independent contribution of the Surpass Elite cannot be determined. The maximum available diameter of 5.25 mm limits its stand-alone use in larger cervical segments. Apposition was assessed on two-dimensional projections and cone-beam CT, without vessel wall imaging, and flow limitation in case 2 was assessed angiographically, without perfusion imaging.

## Conclusions

In two patients with complex cervical ICA dissection, reconstruction with the Surpass Elite flow diverter as part of a hybrid construct was technically feasible, with complete angiographic healing at six months in the patient who reached follow-up. These results cannot be attributed to the device alone, and two cases cannot establish efficacy or superiority over conventional stenting. Antithrombotic therapy remains the standard for most cervical dissections. In selected patients with tortuous, multisegment disease requiring reconstruction, this approach may be considered an alternative that may address some limitations of conventional stenting, provided DAPT preparation is verified with PRU testing.
